# Analysis of cell surface and intranuclear markers on non-stimulated human PBMC using mass cytometry

**DOI:** 10.1371/journal.pone.0194593

**Published:** 2018-03-22

**Authors:** Gaëlle Dzangué-Tchoupou, Aurélien Corneau, Catherine Blanc, Olivier Benveniste, Yves Allenbach

**Affiliations:** 1 Centre of research in Myology, Sorbonne Universités, UPMC Univ Paris 06, INSERM, UMR 974, Pitié-Salpêtrière University hospital, Paris, France; 2 Plateforme de Cytométrie (CyPS), Sorbonne Universités, UPMC Univ Paris 06, INSERM, UMR 1135, Paris, France; 3 Department of Internal medicine and clinical immunology, Pitié-Salpêtrière University hospital, DHU I2B, AP-HP, INSERM, UMR 974, Paris, France; George Mason University, UNITED STATES

## Abstract

Mass cytometry is a powerful tool that allows simultaneous analysis of more than 37 markers at the single cell level. Mass cytometry is of particular interest in the identification of a wide variety of cell phenotypes in autoimmune diseases. Moreover, cells can be labelled with palladium isotopes and pooled before staining (barcoding). Nevertheless, immunologists often face an important problem concerning the choice of markers to be included in a panel. This problem arises due to the incompatibility of different buffers used for the fixation and permeabilization of cells with various cell surface epitopes. In this study, we used a panel of 27 markers (19 surface markers and 8 intranuclear markers) to demonstrate disparities in the detection of cell surface antigens when comparing different buffers to stain unstimulated peripheral blood mononuclear cells. These disparities range from mild differences to very important differences in population frequencies depending on the buffers. Finally, we demonstrate the harmful effects of permeabilization prior to barcoding on the detection of some cell surface antigens. Here, we optimize a protocol that is suitable to use when targeting a large panel including both cell surface and intranuclear markers on unstimulated human peripheral blood mononuclear cells.

## Introduction

Mass cytometry is a powerful innovative cell profiling tool that is based on antigen detection using metal-conjugated antibodies. This approach allows for simultaneous detection of up to 40 markers at the single cell level [1–2]. Moreover, cells can be tagged with palladium isotopes and pooled before staining, thus reducing intra assay variability during the staining of cells and the acquisition of events [3]. The broad detection capacity of cellular targets using mass cytometry is of particular interest to clinical trials, deep phenotyping studies and cell population discovery in various cancers and auto-immune diseases [4]. One of the major challenges encountered when using cytometry is the simultaneous detection of cell surface markers and intranuclear markers. This trouble often arises due to the partial loss of signal intensity of cell surface markers after permeabilization [5]. Consequently, some authors use panels comprised solely of cell surface markers and secreted cytokines [6–8]. Other researchers use permeabilization buffers for the detection of intranuclear markers, but very often this permeabilization is detrimental to cell surface epitopes [9–10]. Either approach ultimately leads to the loss of the complexity and innovative approaches of mass cytometry.

Barcoding samples using palladium isotopes require a quick fixation and permeabilization step. This step can also alter the detection of cell surface markers.

At present, a systematic comparison of the effect of different permeabilization protocols on the visualization of cell surface markers in mass cytometry has never been described. Our aim was to optimize a protocol which allows the detection of a broad panel of cell surface and intranuclear markers on human PBMC (Peripheral Blood Mononuclear Cells).

Here, we used four permeabilization conditions to compare the effects of permeabilization on the detection of a broad panel comprised of cell surface and intranuclear markers using mass cytometry: an adapted BD cytofix/cytoperm protocol, eBioscience permeabilization buffer, MaxPar Nuclear Antigen Staining Buffer (NASB) and Methanol/Paraformaldehyde (PFA). Altogether, cells were labelled with 27 antibodies: 19 antibodies targeting cell surface markers and 8 antibodies targeting intranuclear markers.

## Material and methods

### Clinical samples and storage

Approval for this study was obtained from the *Comité Consultatif sur le Traitement de l’Information en matière de la Recherche dans le domaine de la Santé* (CCTIRS) France. Citrated blood donated by healthy adults was obtained from the Etablissement Français du sang (EFS) at the Pitié Salpêtrière University Hospital. Written informed consent was signed by all donors according to the declaration of Helsinki. Upon reception of blood samples, PBMC were isolated and stored at -80°C in Foetal Bovine Serum (FBS, Life Technologies, Saint-Aubin, France, Catalog # 10270106) supplemented with 10% Dimethyl Sulfoxide. Twenty-four hours later, the cells were transferred to liquid nitrogen until time of use.

### Antibodies and reagents

Twenty-four metal-conjugated antibodies were obtained from Fluidigm (Les Ulis, France). Four purified monoclonal antibodies targeting CD28, CD8, RORγT and Bcl6 were obtained from BD Bioscience (Le pont-de-Claix, France) and conjugated to their respective metal tags as previously described [11]. Briefly, primary antibody transition metal-conjugates were prepared in 200 μg lots with the MaxPAR antibody conjugation kit (Fluidigm, Les Ulis, France) following the manufacturer’s recommendations. After conjugation, antibodies were diluted to a working concentration of 100X in Candor PBS Antibody Stabilization solution (Candor Bioscience GmbH, Le pont Claix, France) and stored at 4°C. The list of antibodies used and their corresponding concentrations are found in **S1 Table**.

### Viability and Iododeoxyuridine (IdU) staining

Cisplatin, IdU, PBS and staining buffer (SB) were obtained from Fluidigm (Les Ulis, France). All centrifugations were performed during 5 minutes at 300x g before permeabilization and at 800x g after permeabilization.

PBMC were rapidly thawed at 37°C in a water bath then washed twice with 10 ml of RPMI-1640 (Sigma-Aldrich, Saint Quentin, France, Catalog # R8758) supplemented with 10% FBS in 50 ml polypropylene conical tubes (Dominique DUTSCHER, Issy-les-Moulineaux, France, Catalog # 352070). Next, the cells were washed in RPMI-1640 alone and stained with IdU (Catalog # 201127) and Cisplatin (Catalog # 201064) as previously described [11]. Briefly, 13 million PBMC were incubated at 37°C for 25 minutes in PBS (Catalog # 201058) containing a final concentration of 50 μM IdU. The cells were washed once with PBS and stained with 5 μM of Cisplatin for 5 minutes at room temperature (RT). Cisplatin was quenched in SB (volume > 5 times the initial volume). Finally, the cells were centrifuged, suspended in 500 ml of SB (Catalog # 201068) and transferred to a 5-ml polypropylene conical tube (Dominique DUTSCHER, Issy-les-Moulineaux, France, Catalog # 2005457).

### Cell surface staining

Five microliters of CCR7 and 7.5 μl of CXCR5 were added to PBMC in a final volume of 75 μl of SB for 15 minutes at RT. Next, 175 μl of a mix containing the other cell surface antibodies were added to the cells for 30 minutes at RT (5 μl of each antibody as well as 7.5 μl of HLADR, IgD, and CD38). Finally, the cells were washed twice with 2 ml of SB and separated into 5 groups for the following protocols:

### Fixation and permeabilization protocols

#### Cell surface staining only

No fixation or permeabilization step was performed. In this manuscript, this experimental condition is termed “CS” in the figure legends.

#### Fixation and permeabilization using BD cytofix/cytoperm buffer (BD, France, Catalog # 554715)

Cells were incubated at RT for 1 hour with BD Cytofix/Cytoperm buffer, then washed twice with BD wash/perm solution diluted 10X in distilled water. Antibodies targeting intranuclear markers were prepared at a final volume of 50 μl in a BD wash/perm solution (1 μl of each antibody, except 1.5 μl of FoxP3) and added to cells for 1 hour at RT. Finally, cells were washed once with 500 μl of the wash/perm solution BD and once with 500 μl of SB. In this manuscript, this experimental condition is termed “ICSb” for intracellular cytokine staining buffer in the figure legends.

#### Fixation and permeabilization using eBioscience Fixation/permeabilization buffer

Fixation/permeabilization buffer concentrate (Catalog #00-5123-43), Fixation/permeabilization buffer diluent (Catalog # 00-5223-56) and permeabilization buffer (Catalog # 00-8333-56) were obtained from eBioscience, Paris, France. The Fixation/permeabilization buffer concentrate was diluted 1:4 with the diluent, and 250 μl was added to cells for 45 minutes at RT. Cells were washed twice with permeabilization buffer diluted 10X in distilled water. Fifty microliters of the mix of antibodies targeting intranuclear nuclear antigens were prepared in permeabilization buffer (1 μl of each antibody, except 1.5 μl of FoxP3) and added to cells for 45 minutes at RT. Finally, the cells were washed twice with SB. In this manuscript, this experimental condition is termed “INSb 1” for intranuclear staining buffer 1 in the figure legends.

#### Fixation and permeabilization using Maxpar Nuclear Antigen Staining Buffer set (NASB) (Fluidigm, les Ulis, France Catalog # 201063)

The cells were treated and stained according to the manufacturer’s instructions. Briefly, Nuclear antigen staining buffer concentrate was diluted 4X in Nuclear antigen staining buffer diluent, and 1 ml was added to cells for 30 minutes at RT. The mix of antibodies targeting intranuclear antigens was prepared in “nuclear antigen staining perm” at a final volume of 50 μl (1 μl of each antibody, except 1.5 μl of FoxP3) and added to cells for 30 minutes at RT. Finally, the cells were washed twice with SB. In this manuscript, this experimental condition is termed “INSb 2” for intranuclear staining buffer 2 in the figure legends.

#### Fixation using Paraformaldehyde (PFA) (Sigma Aldrich, Saint Quentin, France) and permeabilization using and methanol (Sigma Aldrich, Saint Quentin-France)

Two hundred and fifty microliters of 2% PFA were added to cells for 15 minutes at RT. The cells were washed in 1.5 ml of SB and placed on ice for 10 minutes. One millilitre of pre-cooled methanol was added to cells on ice for an additional 10 minutes. The cells were washed in 500 μl of SB, followed by the addition of a mix of 50 μl of antibodies targeting intranuclear markers in SB for 1 hour at RT (1 μl of each antibody, except for 1.5 μl of FoxP3). Two final washes were performed in SB. In this manuscript, this experimental condition is termed “INSb 3” for intranuclear staining buffer 3 in the figure legends.

### Barcoding of samples

Cells from a healthy donor were thawed and stained with Cisplatin as described above. Next, the cells were split into 3 tubes containing 2 million cells each for the following conditions: no barcoding, barcoding before cell surface staining and barcoding after cell surface staining. The samples were labelled with antibodies targeting CD45, CD3, CD4, CD127 and CD25.

#### No barcoding

Antibodies targeting CD45, CD3, CD4, CD127 and CD25 were added to cells at a final volume of 50 μl in SB for 30 minutes at RT as described above.

#### Barcoding before cell surface staining

Cells were incubated in 1 ml of BD cytofix/cytoperm buffer for 10 minutes at RT. Next, cells were washed 2X with 1 ml of BD wash/perm buffer and suspended in 800 μl of BD wash/perm buffer. Palladium isotopes were suspended in 100 μl of each buffer BD wash/perm buffer and added to cells for 30 minutes at RT. Finally, the cells were washed 2X with SB and incubated in a mix of antibodies containing 1 μl of each antibody targeting CD45, CD3, CD4, CD127 and CD25 for 30 minutes at RT as described above.

#### Barcoding after cell surface staining

Cells were incubated in 50 μl of SB containing 1 μl of each antibody targeting CD45, CD3, CD4, CD127 and CD25 for 30 minutes at RT as described above. Next, the cells were incubated with either 1 ml of BD cytofix/cytoperm buffer for 10 minutes at RT. Next, the cells were washed 2X with 1 ml of BD wash/perm buffer and suspended in 800 μl of BD wash/perm buffer. Palladium isotopes were suspended in 100 μl of BD wash/perm buffer and added to cells for 30 minutes at RT. Finally, cells were washed 2X with SB.

### Assessment of the loss of signal intensity of surface markers after barcoding

Cells from a healthy donor were thawed and incubated for 5 minutes in 5 μM Cisplatin as described above. Next, the cells were divided into 3 tubes containing 1 million cells each for no barcode/no permeabilization, permeabilization only/no barcode and permeabilization/barcode groups. Samples were incubated in a final volume of 50 μl SB containing 1 μl of each antibody targeting CD45, CD3, CD4, CD127 and CD25 for 30 minutes at RT as described above.

### DNA staining

After the end of each experimental condition described above, DNA was labelled with Cell-ID Intercalator-Ir-125 μM (Fluidigm, les Ulis, France, Catalog # 201192A) diluted 1:1000 in Maxpar Fix and perm Buffer (Fluidigm, les Ulis, France, Catalog # 201067) overnight at 4°C. Nevertheless, Cell-ID Intercalator-Ir-125 μM was diluted in 2% PFA for the Methanol/PFA permebilization conditions. The following day, the cells were washed once with staining buffer and once with PBS. Immediately before acquisition, cells were washed once with water, filtered and diluted with 4 element EQ beads (Fluidigm, les Ulis, France).

### Mass cytometric data acquisition and analysis

Cellular events were acquired on the Helios, which is available at the “*Plateforme de Cytométrie de la Pitié-Salpêtrière*”. Cells were acquired at a speed of 300 events per second, with a cell length threshold between 10 and 150 pushes. The 4 elements EQ beads were used to normalize files using the Matlab compiler software cell normalizer. Data files were obtained in the FCS file format and analysed using FlowJo software version 10 and Cytobank cloud based platform. Beads were gated out using the following gates: 140/142Ce, 151/153Eu, 165Ho and 175/176 Lu. CD45+ events were selected for the analysis of different cell populations. Cisplatin+ dead cells were gated out and we performed a viSNE (Stochastic Neighbor Embedding) analysis on CD45+ cells for auniform and non-biased separation of events. The settings used for the viSNE run were as follow: equal event sampling (20.000 events each), channels (CD19, CD4, IgD, CD16, CD14, CD8, CD56 and CD3), iterations (1000), perplexity (50) and theta (0.3).

### Statistics

All the experiments were performed at least 3 times independently. Different indivuals were used for independent experiments. For the comparison of frequencies including cell surface markers, the “surface staining only” condition was used as the control. On the other hand, the “eBioscience permeabilization buffer” condition was used as the control for the comparison of intranuclear markers. Statistics were performed using the GraphPad Prism version 6 software. For multiple comparisons, we used one-way ANOVA with Bonferroni’s multiple correction test (* if p<0.05, ** if p<0.001, *** if p<0.0001).

## Results

### Effects of different buffers on the detection of cell surface antigens

**Fig 1:**
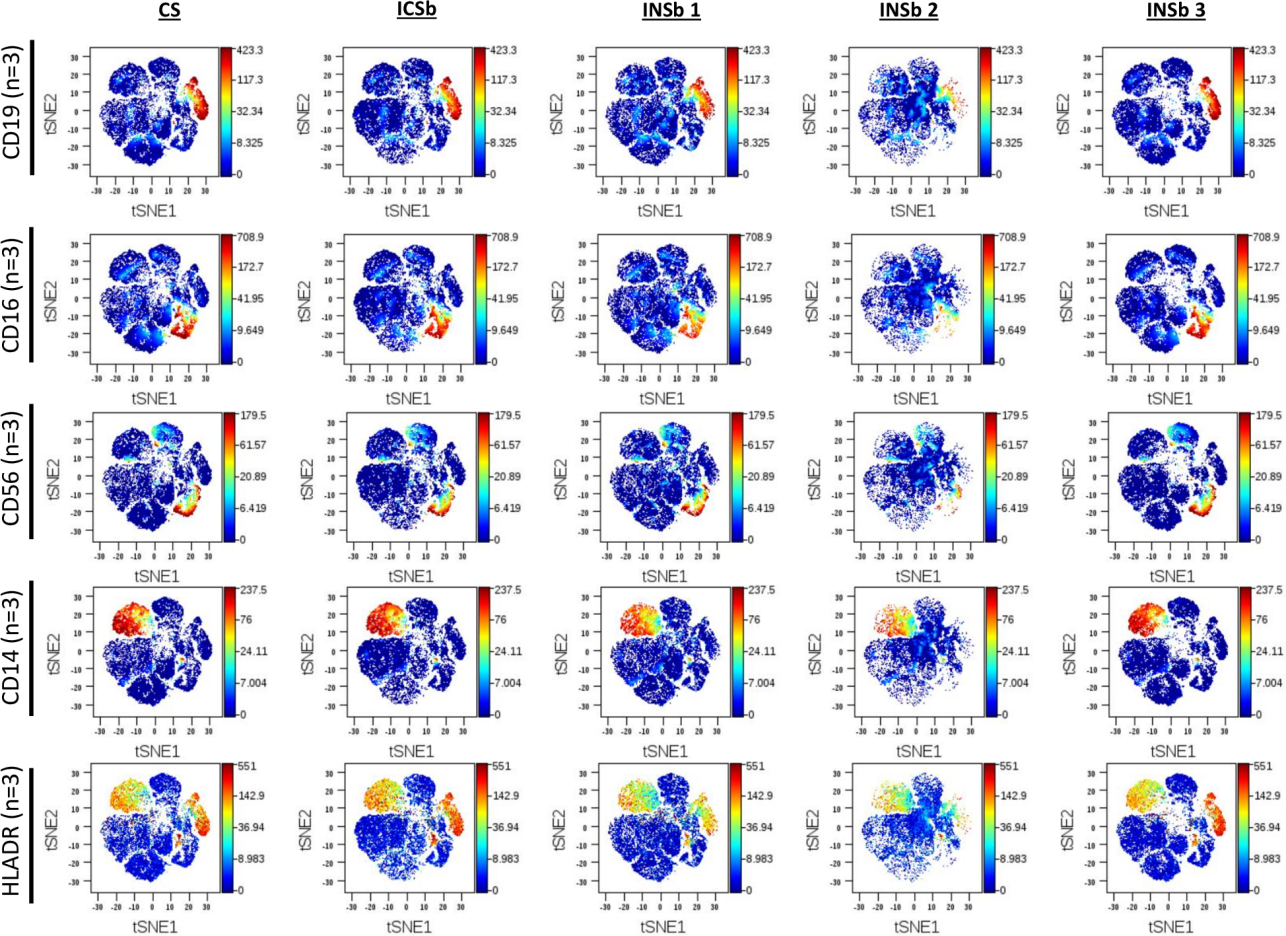
Visualization of the effects of different buffers on the intensity of cell surface markers. Cells from healthy donors were labeled with antibodies targeting both cell surface antigens and intranuclear antigens. Data show the distribution of CD45+ live cells on viSNE plots with the “cell surface staining” only condition (CS) and the different permeabilization conditions: ICSb (BD cytofix/cytoperm buffer), INSb 1 (eBioscience permeabilization buffer), INSb 2 (Maxpar NASB) and INSb 3 (Methanol/PFA). The intensity of CD19+, CD16+, CD56+, CD14+ and HLADR+ events are shown. The concentrations of antibodies used for the detection of these markers are as follows: CD19 (0.5 mg/ml), CD16 (0.2 mg/ml), CD56 (0.1 mg/ml), CD14 (0.3 mg/ml) and HLADR (0.3 mg/ml). viSNE was performed using 1000 iterations, with a perplexity of 30 and theta = 0.3. The data shown are representative of an independent experiment and represent median with interquartile. Experiments were performed 3 times independently. Different healthy individuals were used for each independent experiment.

**Fig 2:**
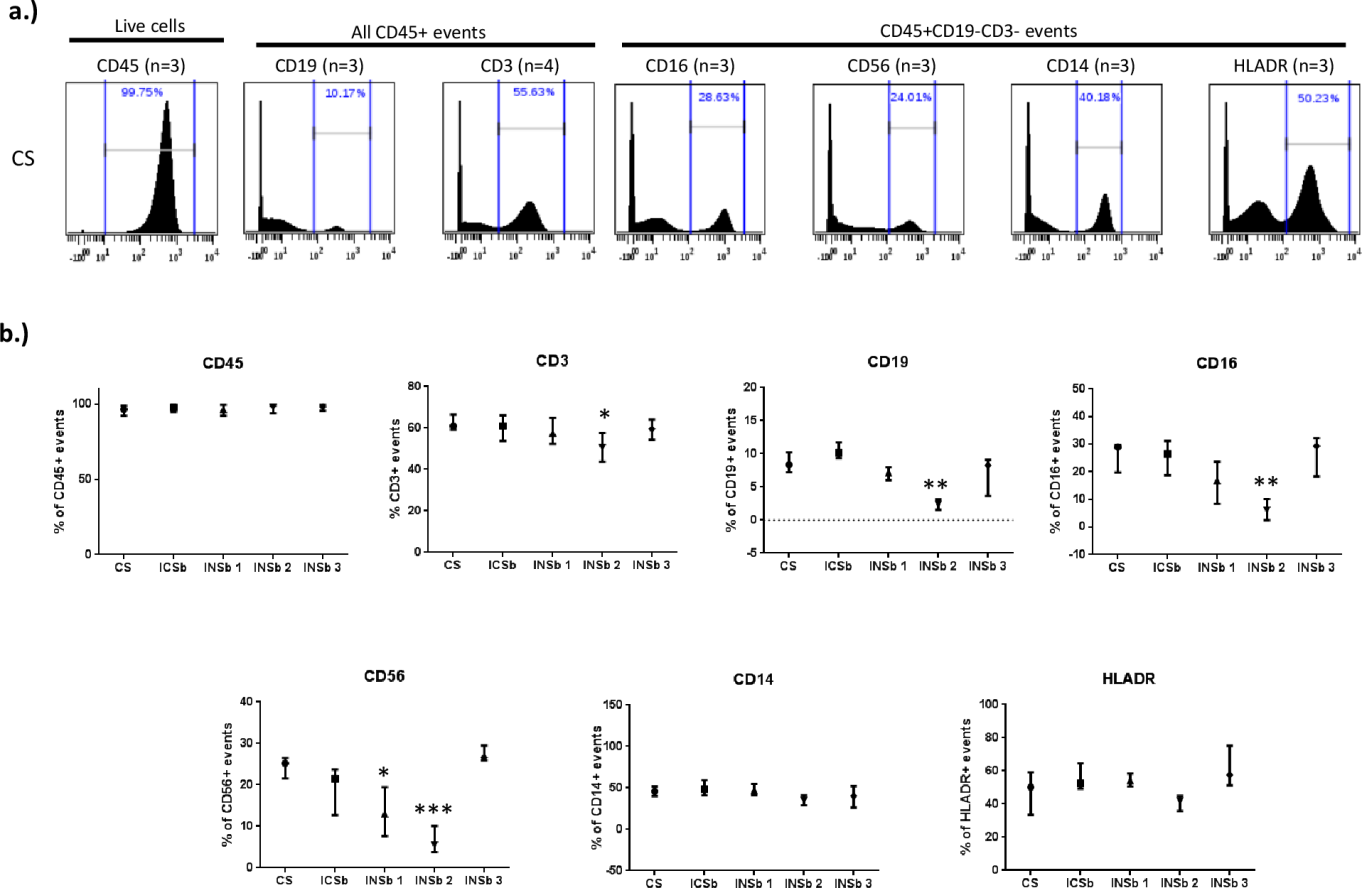
Effects of different buffers on the detection of cell surface markers. Cells from healthy donors were labelled with antibodies targeting both cell surface and intranuclear antigens. Different permeabilization conditions are compared to the cell surface staining only” (CS) condition: ICSb (BD cytofix/cytoperm buffer), INSb 1 (eBioscience permeabilization buffer), INSb 2 (Maxpar NASB) and INSb 3 (Methanol/PFA). Here, we show the effects of different permeabilization conditions on the frequency of various cell surface markers. a.) Histograms showing the frequency and distribution of CD45+, CD19+, CD3+, CD16+, CD56+, CD14+ and HLADR+ events in the CS condition. The concentrations of antibodies used were: CD45 (0.5 mg/ml), CD19 (0.5 mg/ml), CD3 (0.08 mg/ml), CD16 (0.2 mg/ml), CD56 (0.1 mg/ml), CD14 (0.3 mg/ml) and HLADR (0.3 mg/ml). b.) Comparison of the frequency of CD45+, CD19+, CD3+, CD16+, CD56+, CD14+ and HLADR+ events between the CS condition and the different permeabilization conditions. Statistics was performed using one-way ANOVA with Bonferroni’s multiple test correction (*p<0.05, **p<0.001, ***p<0.0001). The data shown are representative of an independent experiment and represent median with interquartile. Experiments were performed 3 times independently. Different healthy individuals were used for each independent experiment.

Our first aim was to compare the effects of different buffers on the detection of cell surface markers. We performed viSNE analyses to avoid bias issues with gating strategy. Strikingly, viSNE plots show variable intensities of surface markers such as CD19, CD56 and CD16 when the “cell surface staining only” conditions were compared to Maxpar NASB and eBioscience Permeabilization buffers (**Fig 1**). Additional viSNE plots showing the intensities of CD45, CD3, CD4, CD8 and IgD are available in **S1 Fig.** Interestingly, BD cytofix/cytoperm buffer and PFA/methanol allowed for detection of population frequencies very close to the “surface staining only” conditions. Greater variations of percentages were observed with eBioscience Permeabilization buffer and Maxpar NASB. For example, the median percentages of CD19+ events, CD16+ events and CD56+ events for all experiments (n = 3–5) is as follows: respectively 9%, 29% and 25% for the control condition, respectively 12%, 26% and 24% for BD cytofix/cytoperm buffer, respectively 8%, 17% and 14% for eBioscience permeabilization buffer, respectively 3%, 7% and 6% for MaxPar NASB and respectively 8%, 29% and 27% for methanol/PFA (**Fig 2**). We performed antibody titration assays, to rule out the possibility that antibodies were not used at saturating conditions **S2 Fig**.

**Fig 3:**
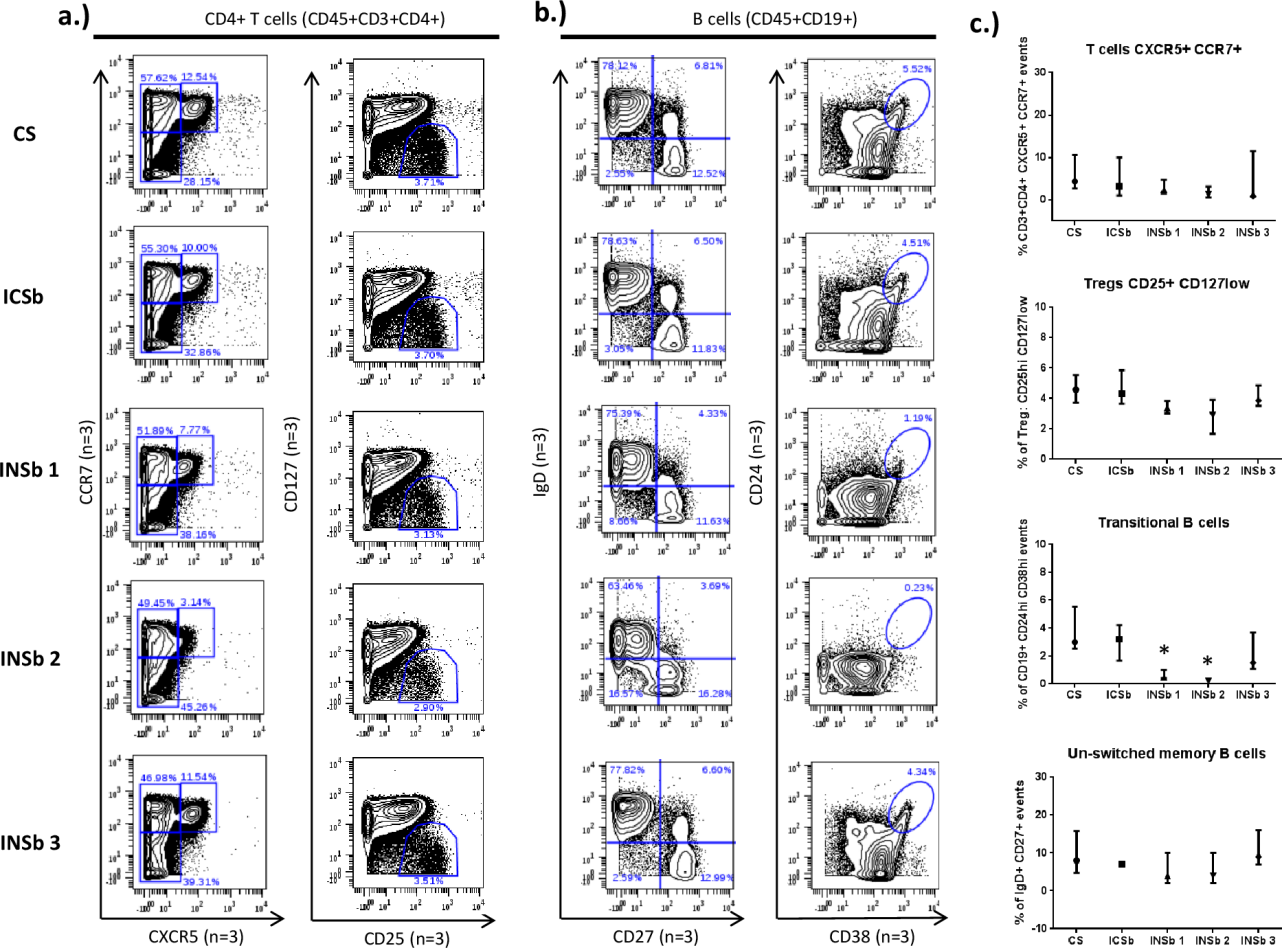
Effects of different buffers on the detection of rare populations. Cells from healthy donors were labelled with antibodies targeting both cell surface and intranuclear antigens. Different permeabilization conditions are compared to the cell surface staining only” (CS) condition: ICSb (BD cytofix/cytoperm buffer), INSb 1 (eBioscience permeabilization buffer), INSb 2 (Maxpar NASB) and INSb 3 (Methanol/PFA). This data compare the frequency of various CD45+ populations between the CS condition and the different permeabilization conditions. a.) Frequencies of rare CD4+ T cell populations: primed T cells (CXCR5+ CCR7+) and Treg cells (CD25^hi^ CD127^low^). b.) Frequencies of rare B cell populations such as transitional B cells (CD24^hi^CD38^hi^) and un-switched memory B cells (CD19+IgD+CD27+). c.) Statistics showing the comparison of the frequency of rare T and B cell populations within the different experimental conditions. The concentrations of antibodies used were: CXCR5 (0.04 mg/ml), CCR7 (0.5 mg/ml), CD25 (0.5 mg/ml), CD127 (0.5 mg/ml), CD24 (0.3 mg/ml), CD38 (0.3 mg/ml), IgD (0.25 mg/ml) and CD27 (0.1 mg/ml). Statistics was performed using one-way ANOVA with Bonferroni’s multiple test correction (*p<0.05, **p<0.001, ***p<0.0001). The data shown are representative of an independent experiment and represent median with interquartile. Experiments were performed 3 times independently. PBMC from different individuals were used for each independent experiment.

Furthermore, variations in the frequencies of rare CD4+ populations, such as regulatory T cells (Treg)(CD3+CD4+CD25^hi^CD127^low^) and primed T cells (CD3+CD4+CXCR5+CCR7+) were observed with eBioscience Permeabilization buffer and Maxpar NASB (**Fig 3**). We also observed a particular distribution of CXCR5/CCR7 events in the PFA/methanol conditions. Finally, we compared the frequencies of rare B cell populations, such as transitional B cells (CD19+CD24^hi^CD38^hi^) and un-switched memory B cells (CD19+IgD+CD27+) among the different conditions (**Fig 3**). The most striking observation was the variation of transitional B cells within the various conditions tested. While the “surface staining only condition”, BD Cytofix/Cytoperm buffer and methanol/PFA conditions displayed a median percentage of 3%, 3% and 1.5% of transitional B cells, respectively, eBioscience Permeabilization buffer and Maxpar NASB displayed only 0.5% and 0.2% respectively (Fig 3). A comparison of the median percentages of naïve B cells (CD19+IgD+CD27-), switched memory B cells (CD19+IgD-CD27+) and other CD4+ T cell populations are available in **S3 Fig.** Histograms of all surface markers are available in **S4 Fig**.

### Detection of intranuclear antigens using different buffers:

**Fig 4:**
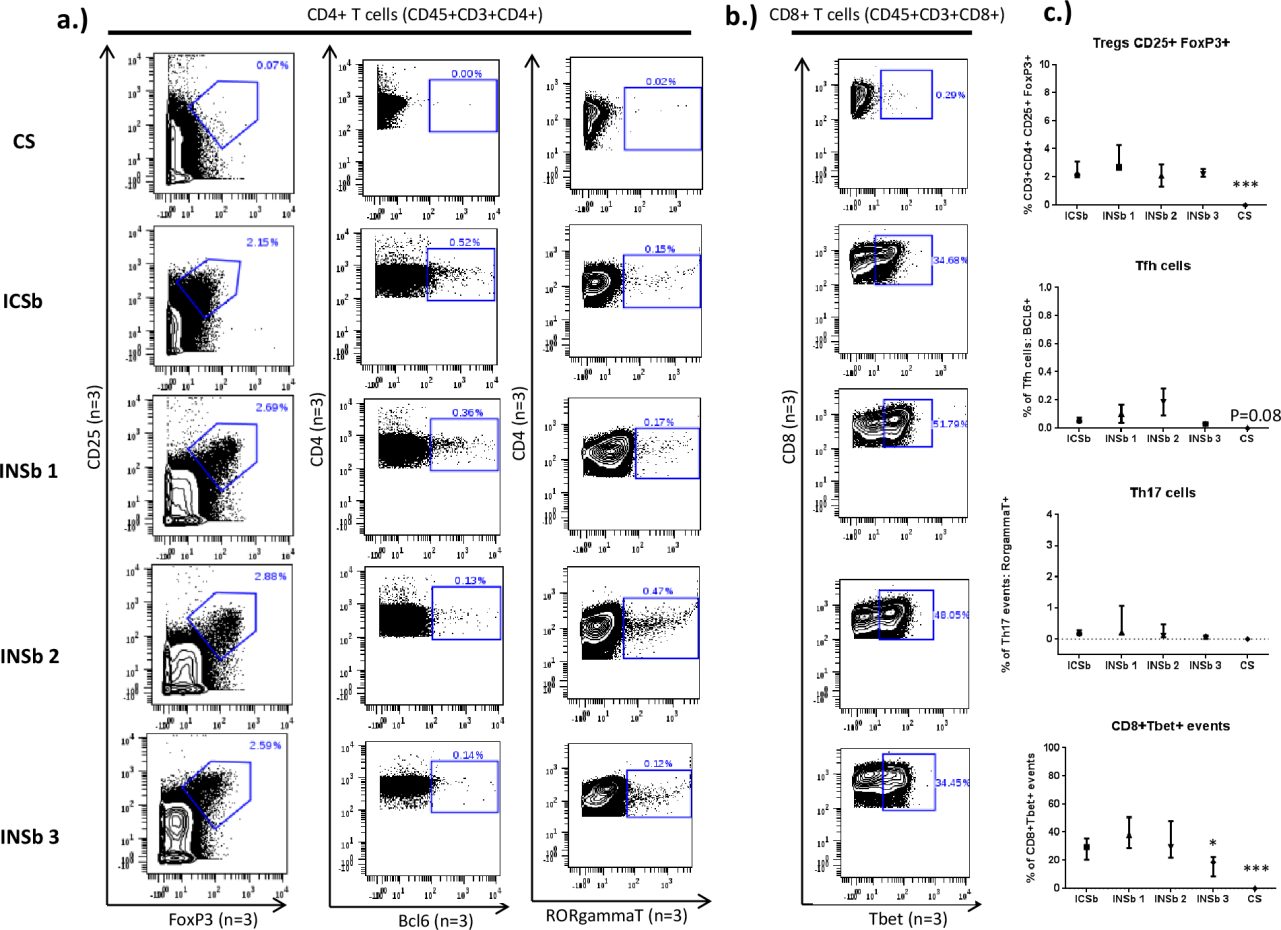
Detection of intranuclear markers using the adapted BD cytofix/cytoperm protocol. Cells from healthy donors were incubated with antibodies targeting cell surface antigens and then split into 5 for the following conditions: the “surface staining only” conditions (CS), and fixation and permeabilization using either BD cytofix/cytoperm buffer (ICSb), eBioscience fixation and permeabilization buffer (INSb 1), Maxpar NASB (INSb 2) and PFA/methanol (INSb 3). Next cells were labelled with a mix of antibodies targeting intranuclear markers. Data compare the frequencies of Treg cells (CD25hiFoxP3+), Tfh cells (CD4+BCL6+), Th17 cells (CD4+RoryT+) and CD8+Tbet+ cells between the various permeabilization conditions. The concentrations of antibodies used are as follow: FoxP3 (0.3 mg/ml), BCL6 (0.8 mg/ml), RoryT (0.6 mg/ml) and Tbet (0.3 mg/ml). Statistics was performed using one-way ANOVA with Bonferroni’s multiple test correction (*p<0.05, **p<0.001, ***p<0.0001). Data obtained from all the other permeabilization conditions were compared to INSb 1 condition. The data shown are representative of an independent experiment and represent median with interquartile. Experiments were performed 3 times independently. PBMC from different individuals were used for each independent experiment.

Our next aim was to compare the effects of the different buffers on the quantification of intranuclear markers. Here, we used different intranuclear markers to define T CD4+ lineages: regulatory T cells (Tregs) (CD3+CD4+CD25^hi^FoxP3+), Th17 cells (CD3+CD4+RorgammaT+), follicular helper T cells (Tfh)(CD3+CD4+Bcl6+) and also CD8+Tbet+ cells. The most striking observation was the detection of intranuclear markers using the adapted BD cytofix/cytoperm protocol. We observed median percentages of 2.7%, 0.2%, 0.1% and 38% respectively for Tregs, Th17 cells, Tfh cells and CD8+Tbet+ cells with eBioscience Permeabilization buffer versus 2.2%, 0.2%, 0.05% and 30% respectively for BD cytofix/cytoperm buffer (**Fig 4**). In addition, the median percentages of Tregs, Th17 cells, Tfh cells and CD8+Tbet+ cells for the Maxpar NASB conditions were: respectively 2.1%, 0.1%, 0.2% and 29% versus 2.2%, 0.05%, 0.03% and 13% respectively for the PFA/Methanol conditions. The adapted protocol using BD cytofix/cytoperm buffer also allowed the detection of cell cycle markers **S4 Fig.**

### Effects of permeabilization prior to barcoding on the detection of cell surface antigens

**Fig 5:**
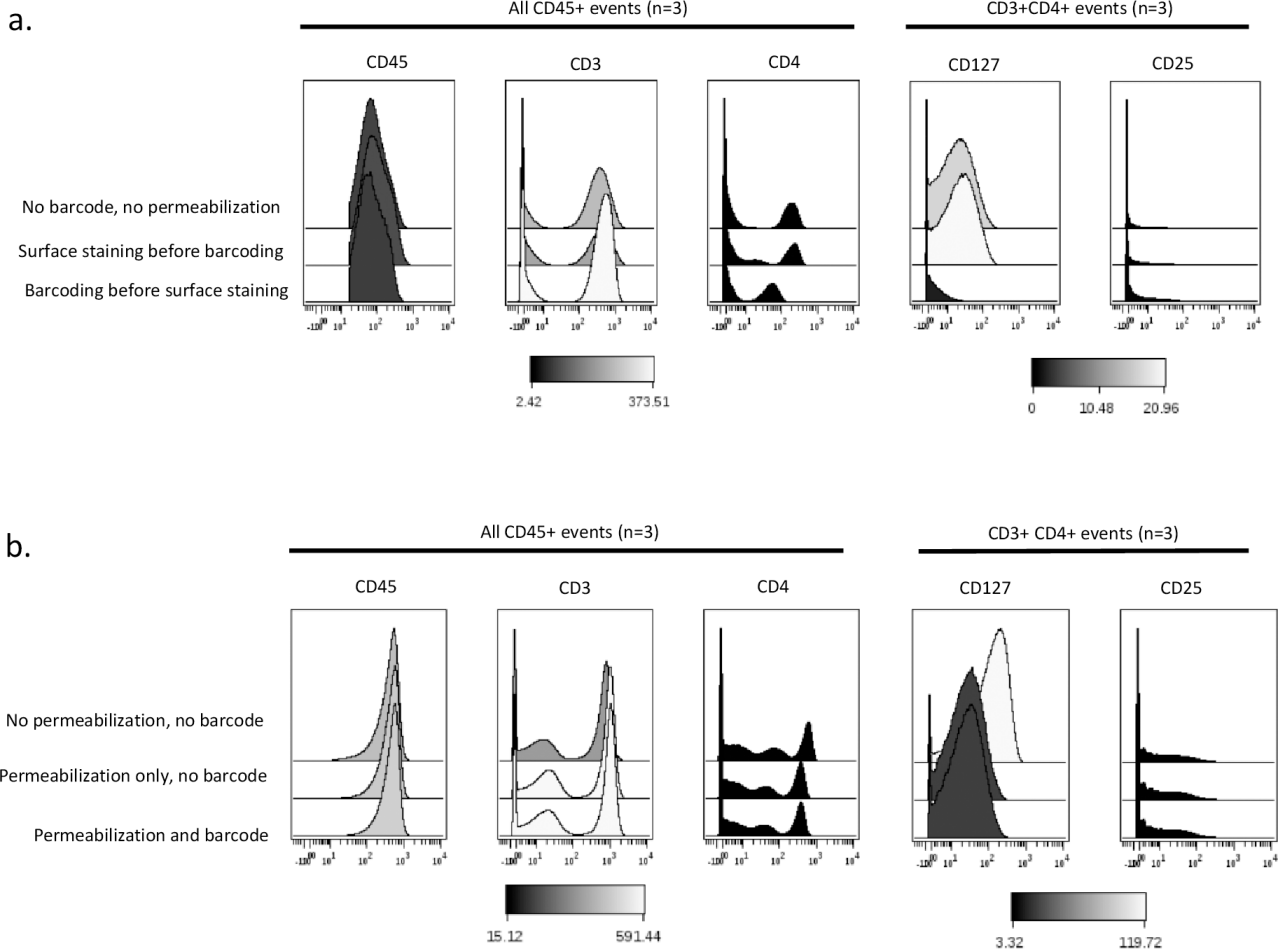
Partial loss of the signal intensity of CD4 and CD127 after barcoding. Cells were thawed as described above and used for the following purposes: a) assessment of the effects of barcoding on the expression of surface markers. Three experimental conditions were performed: no barcode/no permeabilization, surface staining before barcoding and finally barcoding before surface staining. Data show histogram overlays of CD45, CD3, CD4, CD127 and CD25 for the different conditions. The concentrations of antibodies used are as follow: CD45 (0.5 mg/ml), CD3 (0.08 mg/ml), CD4 (0.25 mg/ml), CD127 (0.5 mg/ml) and CD25 (0.5 mg/ml). b) Assessment of the cause of the lower signal intensity of CD4 and CD127 when PBMC are barcoded before cell surface staining. 3 conditions were evaluated: no barcode/no permeabilization, permeabilization only/no barcoding and permeabilization followed by barcoding. Data show histogram overlays of CD45, CD3, CD4, CD127 and CD25 for the different conditions. The data shown are representative of an independent experiment. Experiments were performed 3 times independently. PBMC from different individuals were used for each independent experiment.

Finally, we assessed the effects of the fixation and permeabilization step prior to barcoding on the signal intensity of 5 surface antigens (CD45, CD3, CD4, CD127 and CD25). We observed a very weak signal intensity of CD127 and a loss of the CD4^intermediate^ populations when barcoding was performed prior to cell surface staining. What remained unclear was whether the barcode or the permeabilization step was responsible for this weaker expression. Surprisingly, the weaker signal intensity of CD4 and CD127 was observed when BD permeabilization buffer was used alone without any barcode (**Fig 5**). Increase concentrations of CD127 and CD4 antibodies were not sufficient to prevent the loss observed (**S6 Fig**).

## Discussion

Mass cytometry is an innovative tool that allows for simultaneous detection of up to 40 markers at the single cell level. The broad detection capacity of mass cytometry is of particular interest when a wide range of cellular markers can be identified at the same time, which makes this tool a precious asset in clinical trials, deep phenotyping and cell population discovery [12–13]. Nevertheless, it is important to choose appropriate fixation and permeabilization conditions to clearly detect cell surface and intranuclear antigens. In this study, we assessed the effects of 4 permeabilization conditions on the detection of cell surface and intranuclear markers on non-stimulated human PBMC: “BD cytofix/cytoperm buffer”, “eBioScience permeabilization buffer”, “Maxpar NASB” and “PFA/Methanol”. We used a panel of 27 antibodies including 19 antibodies targeting cells surface antigens and 8 antibodies targeting intranuclear antigens.

The frequencies of the populations described in the control conditions are in accordance with those observed in the literature [14–17]. As expected, permeabilization was associated with lower frequencies of populations gated using surface markers. The variations observed were dependent on the buffer used. Variations in antibody concentrations are ruled out, since all cells were stained together before being split into the different permeabilization conditions. Considering that antibodies are efficiently coupled to surface antigens before the fixation/permeabilization step, we believe that the variations observed are due to the partial destruction of epitopes by the buffers.

Epitope instability upon permeabilization is well known in flow cytometry [5]; which explains our observations. Nevertheless, mass cytometry offers a broader range of antigen detection compared to flow cytometry. Therefore, the setting of a suitable protocol to limit epitope instability is necessary to optimize the potential of mass cytometry.

For permeabilization protocols, we choose as reference eBioscience buffer, since the results we obtained (intranuclear T CD4+ cells markers) were similar with those previously reported [18–20]. It is important to remind that BD cytofix/cytoperm is a buffer for intracellular cytokines detection, whereas MaxPar NASB and Methanol/PFA are dedicated to intranuclear staining. In this study, the optimization we made of BD cytofix/cytoperm protocol permits the detection of intranuclear targets with the same efficacy as intranuclear permeabilization buffers (eBioscience Permeabilzation buffer, MaxPar NASB and PFA/Methanol). In addition, this optimized protocol does not alter the detection of surface markers.

Here, we also assessed the effects of the recommended quick permeabilization step prior to barcoding on the detection of 5 surface markers. We observed weaker signal intensities of CD4 and especially CD127 when cells were barcoded before cell surface staining. Accordingly, the poor performance of CD127 upon barcoding was previously described [7]. To overcome such issues, Zunder and colleagues described various troubleshooting approaches when barcoding cells [3]. They described the detection of lower antibody staining intensity as a result of high mass-cell tag barcoding. In this study, we demonstrated that the lower staining intensity of CD127 and CD4 is not due to the mass-cell barcoding tag, but rather to the permeabilization step performed. Recently, a different barcoding approach based on CD45 coupled to different metal tags was described [21–22]. This novel barcoding approach does require any permeabilization step; however its application is limited to cells that uniformly express CD45.

## Conclusion

In conclusion, here we compared different permeabilization protocols and showed that our adapted protocol allows the detection of intranuclear markers without altering the detection of a large panel of cell surface markers. Finally, we also confirmed the poor performance of CD127 upon barcoding. We show that this poor performance is due to the permeabilization step, and not due to the palladium isotopes. We suggest performing barcoding after cell surface staining or after the staining of sensitive epitopes, thereby protecting sensitive epitopes while preserving a uniform intra-assay staining and acquisition of events.

## Supporting information

S1 Table List of antibodies. (PDF)

S1 Fig Distribution of CD45, CD3, CD4, CD8 and IgD on viSNE plots. (PDF)

S2 Fig Saturating conditions of CD19, CD3, CD16 and CD56. (PDF)

S3 Fig Median percentages of B and T cell populations. (PDF)

S4 Fig Histograms of all surface markers. (PDF)

S5 Fig Visualization of cell cycle markers. (PDF)

S6 Fig Saturating conditions of antibodies for barcoding conditions. (PDF)
