# Supplementary material for: Analysis of cell surface and intranuclear markers on non-stimulated human PBMC using mass cytometry
Source: PLoS One. 2018 Mar 22;13(3):e0194593. doi: 10.1371/journal.pone.0194593 (PMC5864033; doi:10.1371/journal.pone.0194593)
Supplement: S1 Table — (PDF) [file pone.0194593.s001.pdf]

S1 Table. List of antibodies.

Antigen	Metal tag	Manufacturer	Catalog #	Lot #	Clone	Concentration (mg/ml)
		Cell surface markers				
CD45	89	Fluidigm	3089003B	2991609	HI30	0,5
CD19	142	Fluidigm	3142001B	0331503	HIB19	0,5
HLADR	143	Fluidigm	3143013B	1631407	L243	0,3
CD4	145	Fluidigm	3145001B	2171601	RPA-T4	0,25
IgD	146	Fluidigm	3146005B	0621515	IA6-2	0,25
CD16	148	Fluidigm	3148004B	0871423	3G8	0,2
CD14	151	Fluidigm	3151009B	2101406	M5E2	0,3
CD28	154	BD Pharmingen	555725	4311676	CD28.2	0,59
CD27	158	Fluidigm	3158010B	0421404	L128	0,1
CCR7	159	Fluidigm	3159003A	1891504	G043H7	0,5
CD8	161	BD Pharmingen	555363	89931	PURIFIED NA/LE MOUSE	0,63
CD56	163	Fluidigm	3163007B	0131506	NCAM16.2	0,1
CD24	166	Fluidigm	3166007B	1431401	ML5	0,3
CD138	168	Fluidigm	3168009B	0031406	DL101	0,5
CD25	169	Fluidigm	3169003B	1071401	2A3	0,5
CD3	170	Fluidigm	3170001B	1921405	UCHT1	0,075
CXCR5	171	Fluidigm	3171006B	1141316	51505	0,04
CD38	172	Fluidigm	3172007B	0191505	HIT2	0,3
CD127	176	Fluidigm	3176004B	1071409	A019D5	0,5
		Intranuclear markers				
IdU	127	Fluidigm	201127			50mM
phosphoRb	150	Fluidigm	3150013A	0911407	J112-906	0,25
Tbet	160	Fluidigm	3160010B	0141528	4B10	0,3
FOXP3	162	Fluidigm	3162024A	1591502	259D/C7	0,3
CyclinB1	164	Fluidigm	3164010A	3031309	GNS-1	0,25
RORγT	173	BD Pharmingen	562197	13889	028-835	0,56
phosphoH3	175	Fluidigm	3175012A	0871420	HTA28	0,3
		DNA markers				
DNA1	191	Fluidigm	201192A	0891001		
DNA2	193	Fluidigm	201192A	0891001		
