# Supplementary figures and images for: Analysis of cell surface and intranuclear markers on non-stimulated human PBMC using mass cytometry
Source: PLoS One. 2018 Mar 22;13(3):e0194593. doi: 10.1371/journal.pone.0194593 (PMC5864033; doi:10.1371/journal.pone.0194593)

**S1 Fig. Distribution of CD45, CD3, CD4, CD8 and IgD on viSNE plots.**

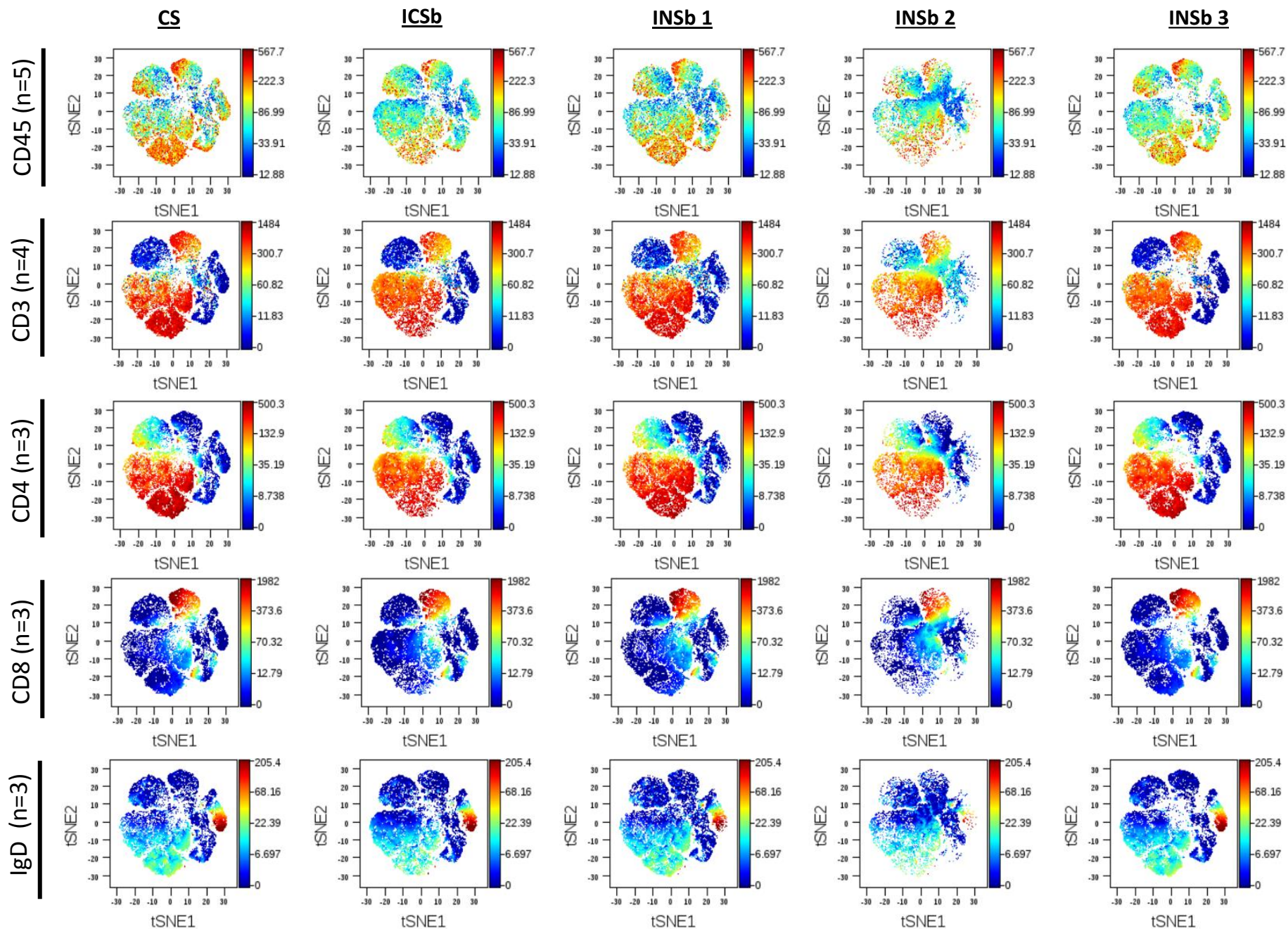

Supplement: S1 Fig — (PDF) [file pone.0194593.s002.pdf]

## S2 Fig. Saturating conditions of CD19, CD3, CD16 and CD56.

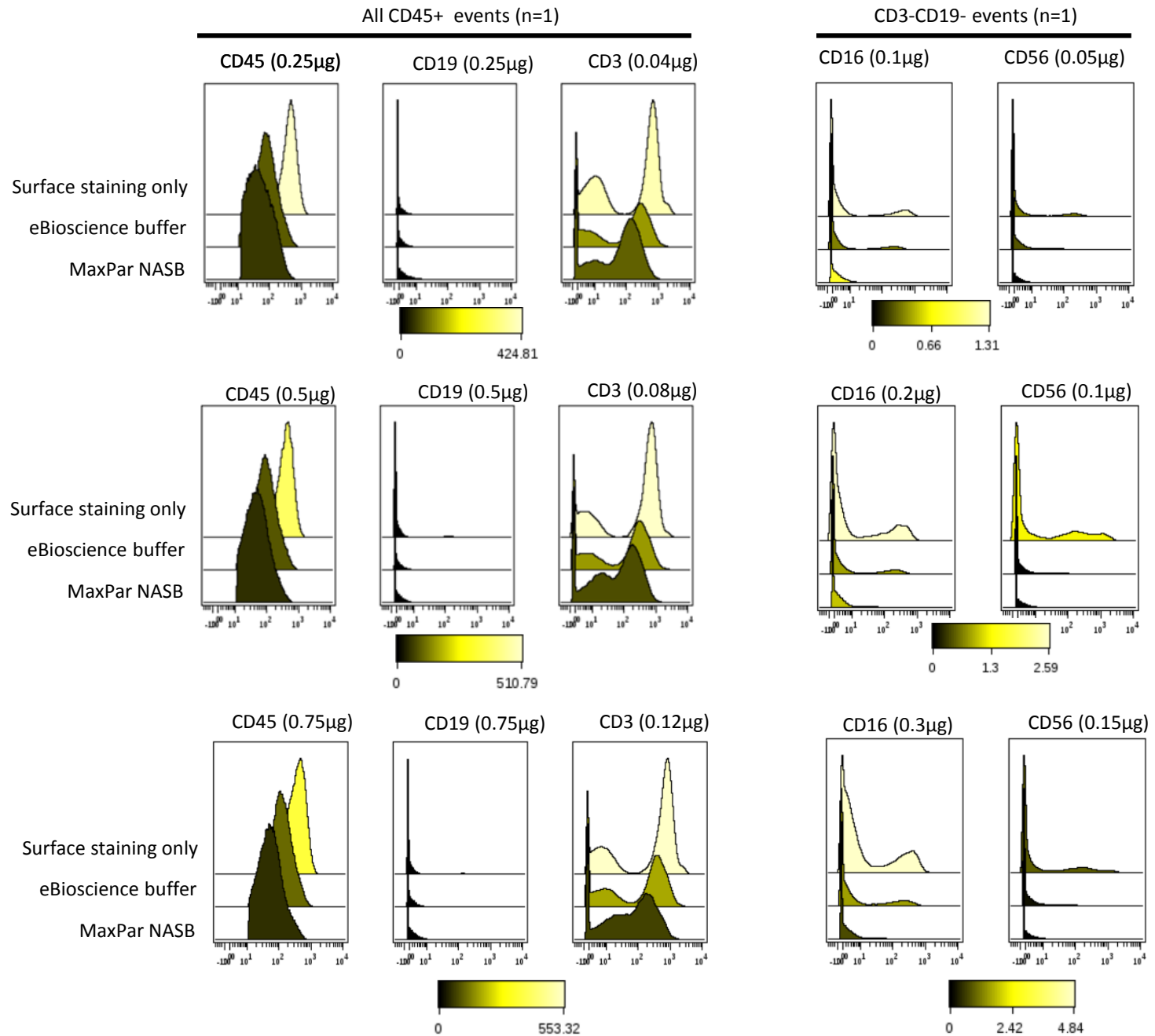

Supplement: S2 Fig — (PDF) [file pone.0194593.s003.pdf]

**S3 Fig. Median percentages of B and T cell populations.**

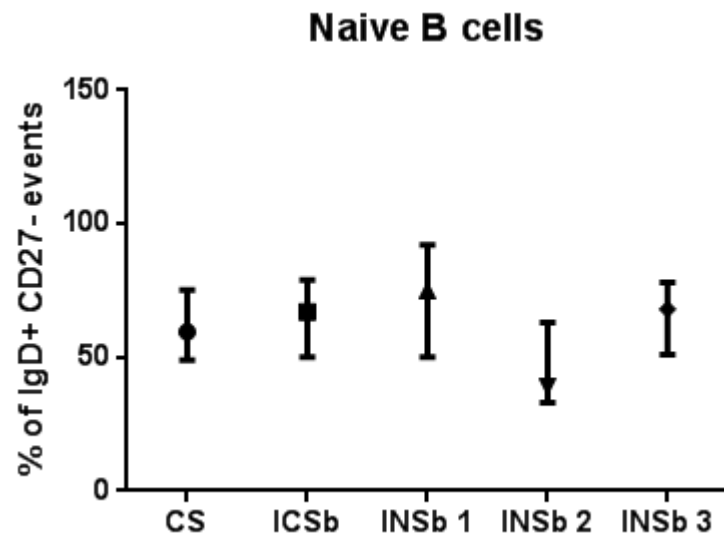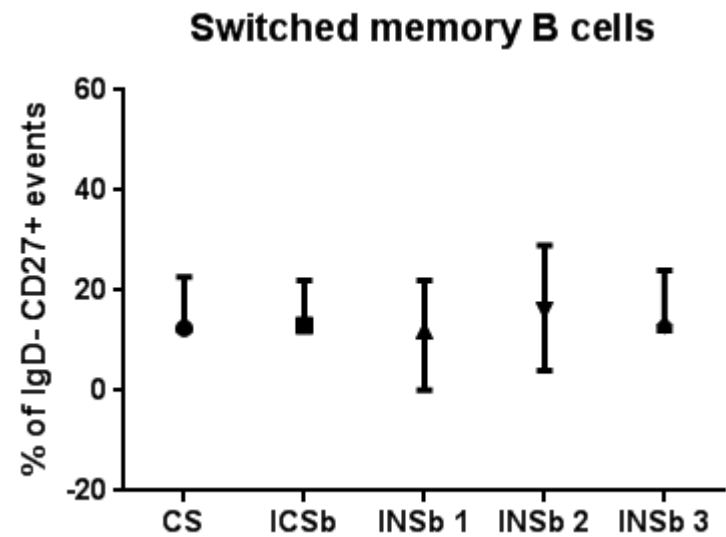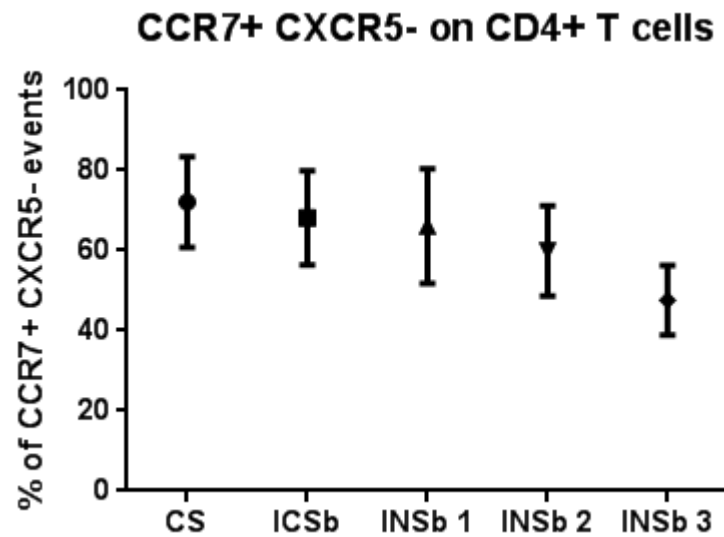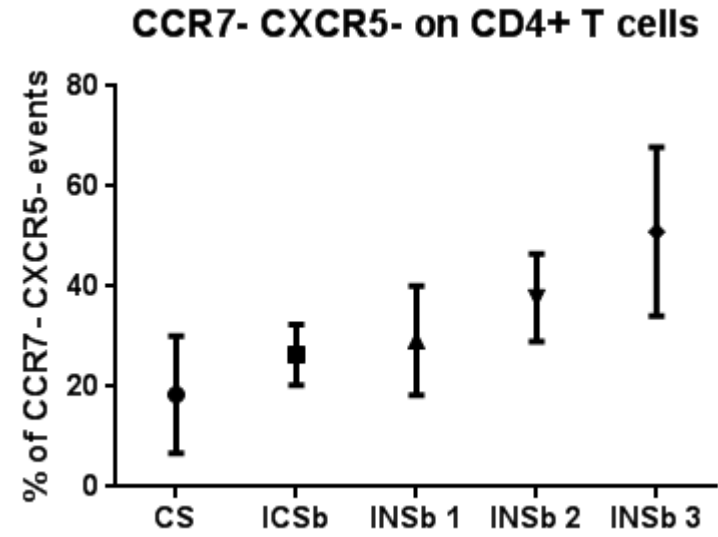

Supplement: S3 Fig — (PDF) [file pone.0194593.s004.pdf]

# S4 Fig. Histograms of all surface markers.

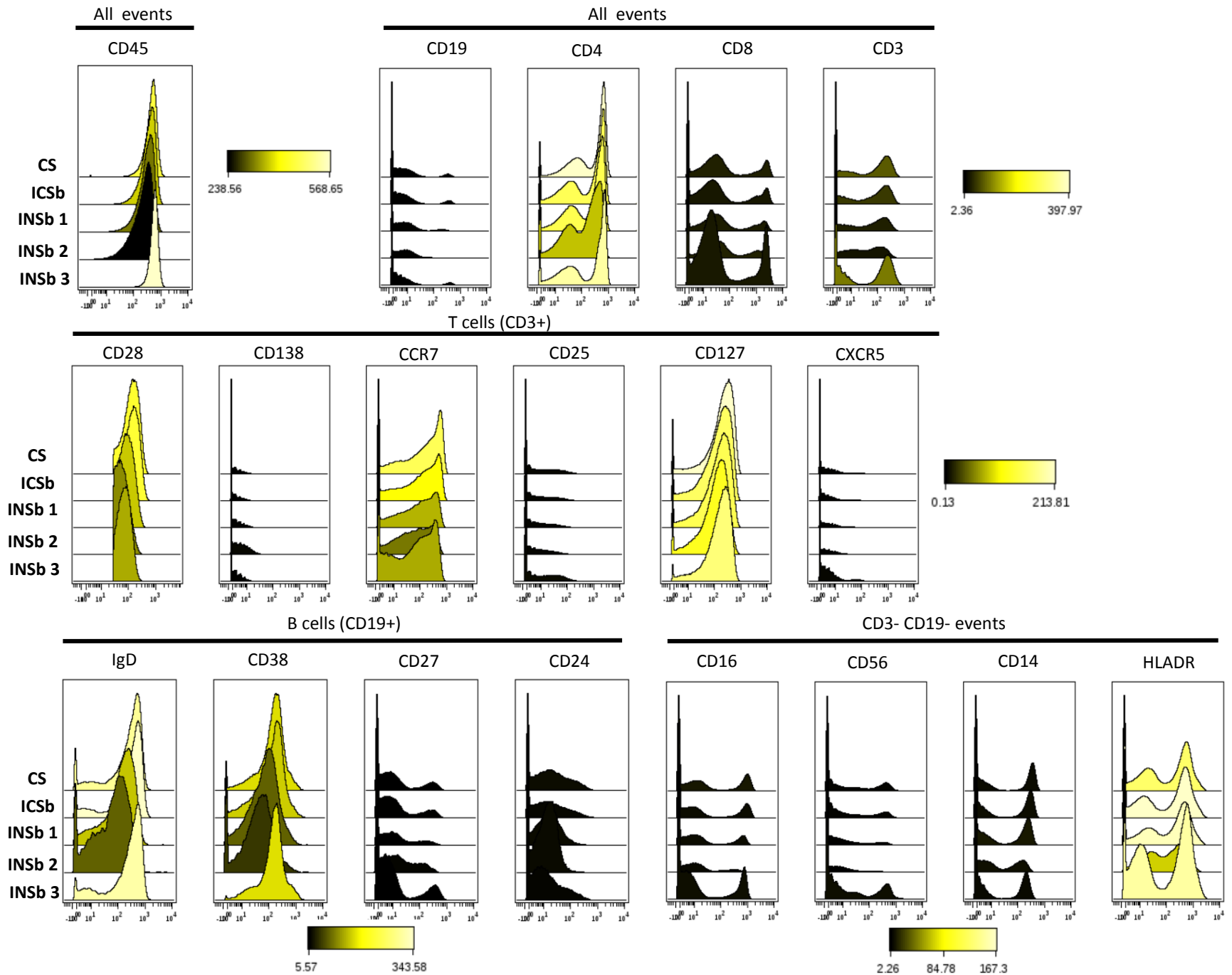

Supplement: S4 Fig — (PDF) [file pone.0194593.s005.pdf]

# S5 Fig. Visualization of cell cycle markers.

T cells (CD3+)

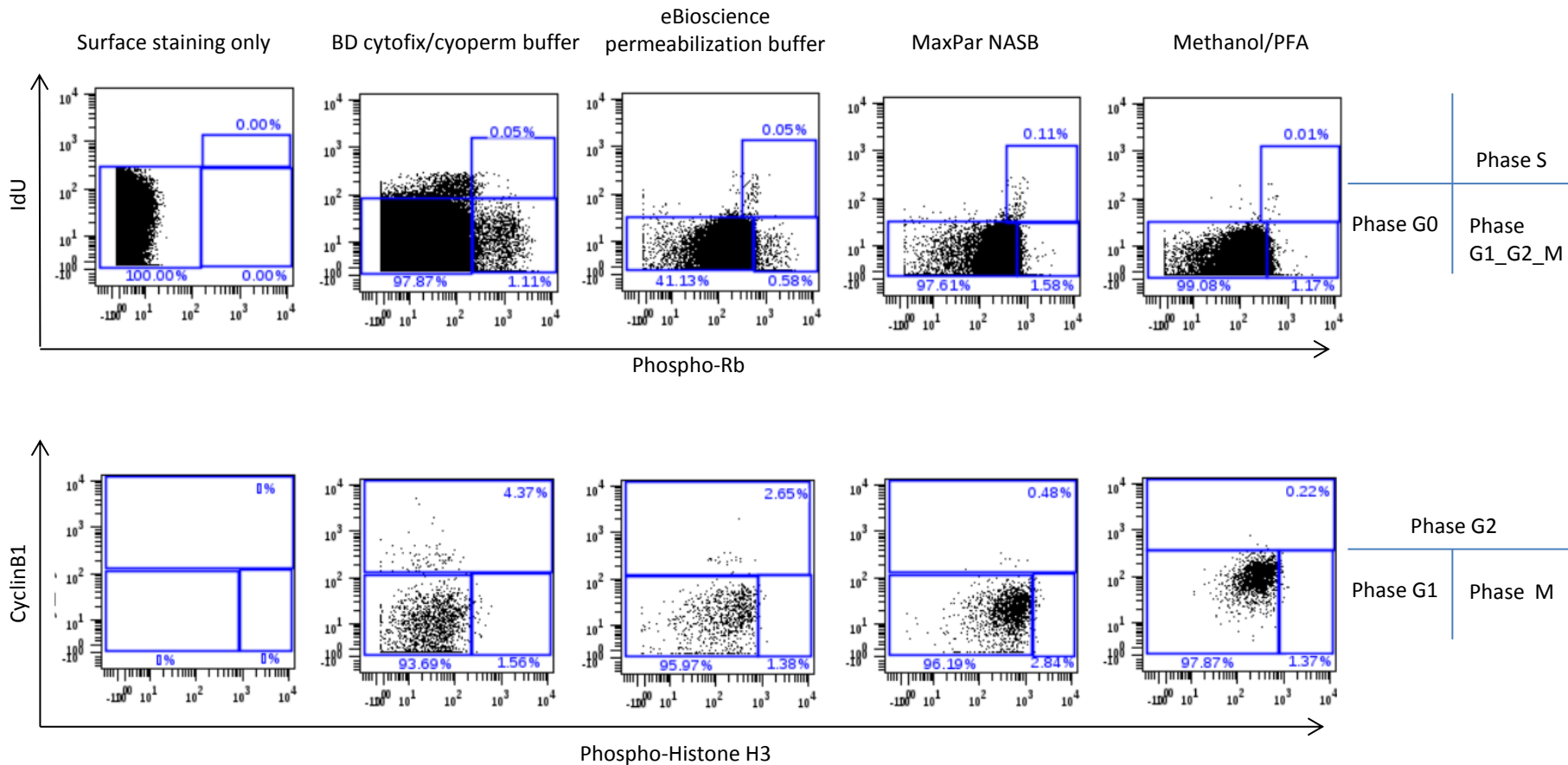

n=3 for all markers

Supplement: S5 Fig — (PDF) [file pone.0194593.s006.pdf]
