# Supplementary material for: Analysis of cell surface and intranuclear markers on non-stimulated human PBMC using mass cytometry
Source: PLoS One. 2018 Mar 22;13(3):e0194593. doi: 10.1371/journal.pone.0194593 (PMC5864033; doi:10.1371/journal.pone.0194593)

S6 Fig. Saturating conditions of antibodies for barcoding conditions.

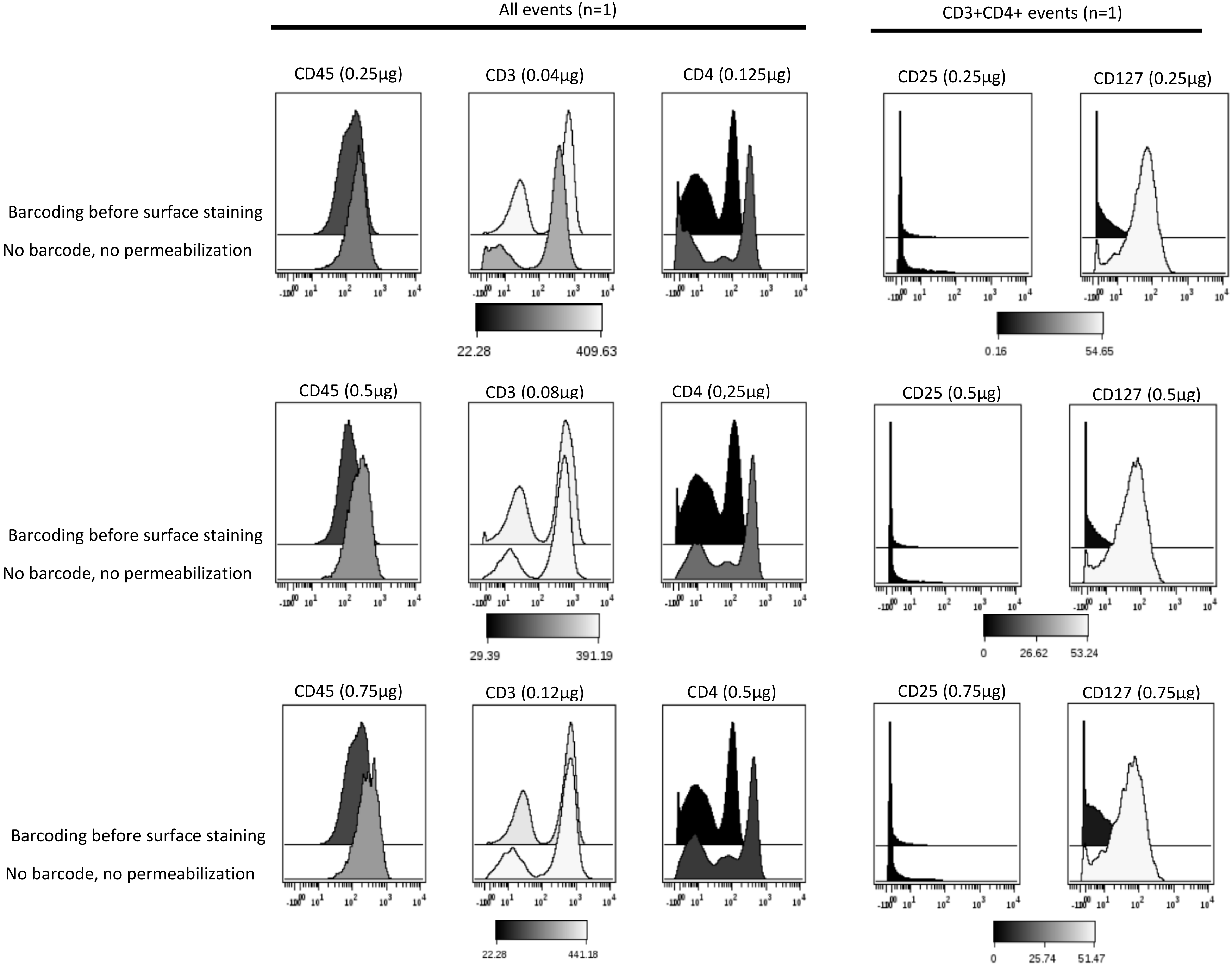

Supplement: S6 Fig — (PDF) [file pone.0194593.s007.pdf]
